# A potent Vip3Aa94 protein from Thai *Bacillus thuringiensis*: molecular characterization and insecticidal potential

**DOI:** 10.7717/peerj.21207

**Published:** 2026-05-27

**Authors:** Ratchadawan Ngoen-Klan, Naruemon Chinon, Atsalek Rattanawannee, Atirach Noosidum, Wanwisa Siriwan, Jariya Chanpaiseang, Theeraphap Chareonviriyaphap, Prakai Rajchanuwong

**Affiliations:** 1 Research and Lifelong Learning Center on Urban and Environmental Entomology, Kasetsart University, Bangkok, Thailand; 2 Department of Entomology, Faculty of Agriculture, Kasetsart University, Bangkok, Thailand; 3 Department of Plant Pathology, Faculty of Agriculture, Kasetsart University, Bangkok, Thailand

**Keywords:** Biocontrol agent, *Bacillus thuringiensis*, Vip3 gene, Insecticidal activity, *Spodoptera* spp.

## Abstract

Vegetative insecticidal proteins (Vip) are produced by *Bacillus thuringiensis* during its vegetative growth stage. Vip3 proteins play a significant role in the insecticidal activity of *B. thuringiensis* against a wide spectrum of economically important crop pests. In this study, 163 *B. thuringiensis* strains were screened for the presence of *vip3* using a polymerase chain reaction-based approach. Seventeen (10.43%) strains yielded an amplification product for *vip3*. Ten of these strains exhibited larvicidal activity against second-instar larvae of *Spodoptera litura*, * Spodoptera exigua*, and *Spodoptera frugiperda*, with corrected mortality rate ranging from 18.30% to 100%. JC20 exhibited the highest efficacy against the three *Spodoptera* species, achieving corrected mortality rates of 98.30% for *S. litura* and both 100% for *S. exigua* and *S. frugiperda*. Given its potency, JC20 was further characterized to identify its unique features and to assess its potential as a bioinsecticide. Scanning electron microscopy revealed the presence of bipyramidal crystal proteins in JC20, correlating with this strain harboring *cry1D* and *cry2A*. The *vip3* gene of JC20 was cloned and sequenced. The complete 2,370 bp sequence was subsequently named Vip3Aa94 by the Bacterial Pesticidal Protein Database. The size of Vip3Aa94, determined via sodium dodecyl sulfate-polyacrylamide gel electrophoresis was approximately 90 kDa. Broad-spectrum efficacy for Vip3Aa94 was established against the second—instar of *S. frugiperda*, *S. exigua,* and *S. litura*, with low LC_50_ values of 67.38, 81.97, and 147.92 ng/cm^2^, respectively. Phylogenetic analysis based on the Vip3Aa94 sequence indicated that geographic separation led to the divergence of* vip3*, consistent with its distinct features compared with other *B. thuringiensis* strains globally. This study highlights JC20, with its high insecticidal activity, as a promising candidate for controlling *Spodoptera* pests.

## Introduction

The genus *Spodoptera* includes some of the most significant insect crop pest species that are highly polyphagous. The larvae of these insects feed on more than 100 host plants, including rice, maize, cotton, and various vegetables (Goergen et al., 2016; Cheng et al., 2017). *Spodoptera exigua* (Hübner), *Spodoptera frugiperda* (J.E. Smith), and *Spodoptera litura* (Fabricius) are the three most important crop pest species in this genus. *Spodoptera frugiperda*, commonly known as the fall armyworm, is an invasive pest that was first detected in Thailand in 2018 (IPPC, 2018). It causes 25–40% reduction in maize yield, potentially increasing the country’s overall production costs by an estimated 26–52 million USD per annum (Thirawut et al., 2023).

The extensive use of insecticides to control agricultural pests often leads to the contamination of crops with toxic residues, harm to non-target organisms, and development of insecticide resistance in insect pests (Che et al., 2013; Hilliou et al., 2021). As an alternative, microbial insecticide products based on spores and crystal proteins of the bacterium *Bacillus thuringiensis* (*Bt*) have been used for years to control insect pests (Fiuza et al., 2017). *Bt* produces a diverse array of insecticidal proteins, including crystal (Cry) proteins, cytolytic (Cyt) proteins, and secreted insecticidal proteins such as vegetative insecticidal proteins (Vip), which act against the larvae of various insect species (Crickmore et al., 2021). Vip proteins are synthesized by *Bt* during the vegetative growth and sporulation phases, and are subsequently secreted into the culture medium (Chakroun et al., 2012). Based on their amino acid sequence similarity, these proteins are classified into four families: Vpb1, Vpa2, Vip3, and Vpb4 (Crickmore et al., 2021). The Vip3 family comprises three subfamilies, Vip3A, Vip3B, and Vip3C, which are recognized for their potent insecticidal activity, especially against a wide range of lepidopterans (Estruch et al., 1996; Palma et al., 2013).

Studies on the insecticidal properties of Vip3 have primarily focused on Vip3A, which shows broad-spectrum activity against lepidopteran pests (Milne et al., 2008) by interacting with more receptors in the midgut than those targeted by Cry proteins (Lee, Miles & Chen, 2006; Liu et al., 2011; Gouffon et al., 2011), leading to pore formation (Lee et al., 2003) and subsequent cell lysis (Yu et al., 1997). This unique mode of action makes Vip3A a valuable tool for managing insect resistance, either independently or in combination with Cry proteins.

Since the identification of Vip3Aa, researchers have extensively screened *Bt* collections for novel *vip3* genes, and have greatly expanded the *vip3* family with many new members (Loguercio et al., 2002; Mesrati, Tounsi & Jaoua, 2005; Hernández-Rodríguez et al., 2009; Palma et al., 2013; Sauka & Benintende, 2017; Hemthanon, Promdonkoy & Boonserm, 2023). However, information regarding *vip3* genes in Thai *Bt* collections remains limited. This study was aimed at screening and identifying *vip3* genes from *Bt* strains collected in Thailand and evaluating their potential as biocontrol agents with high larvicidal activity against *S. exigua*, *S. litura*, and *S. frugiperda* based on their biochemical properties, morphological traits, protein profiles, and *cry* gene content.

## Materials & Methods

### *Bacillus thuringiensis* strains

**Figure 1:**
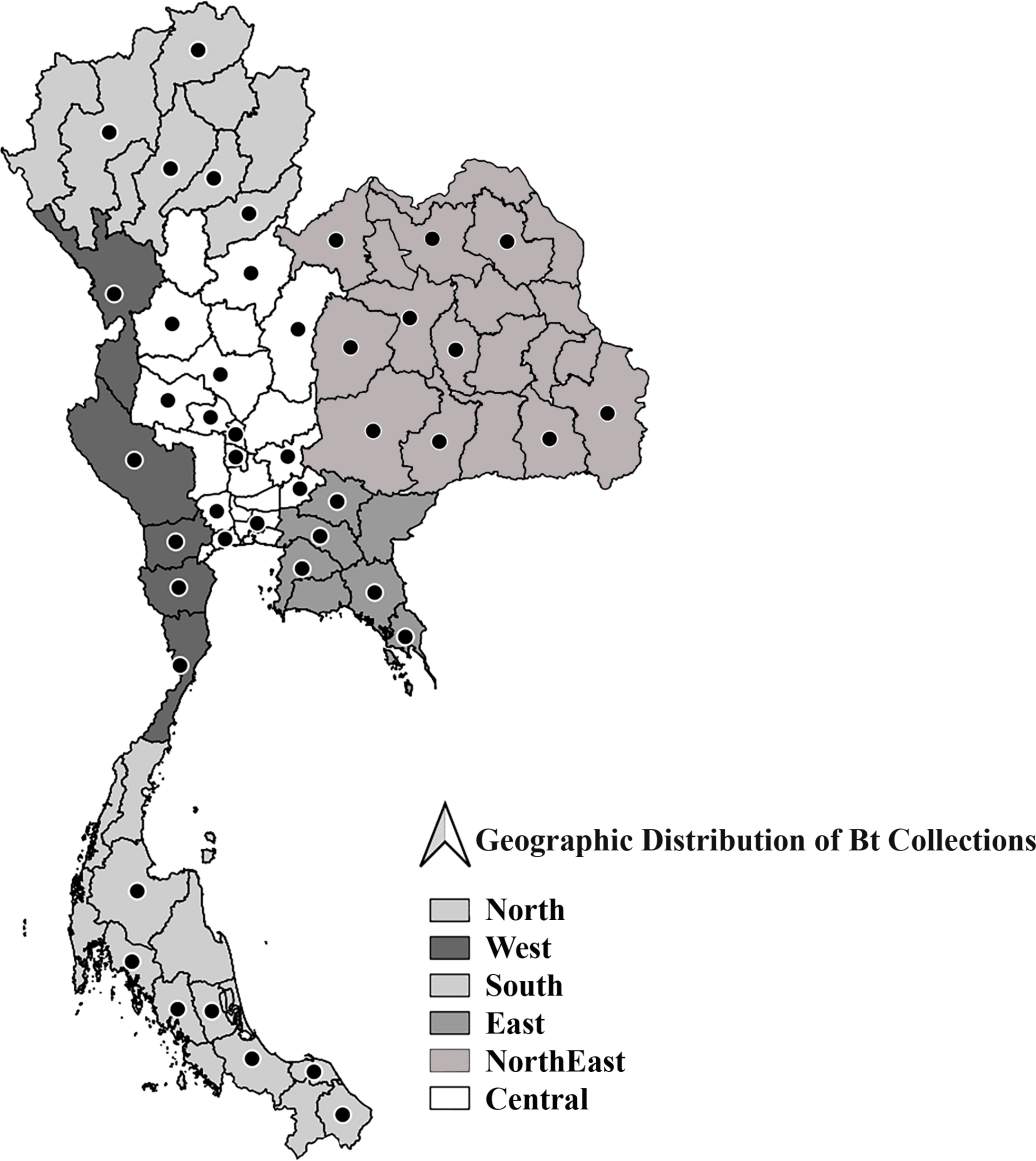
Geographical origins of *Bacillus thuringiensis* strains in Thailand. Thailand boundary was obtained from Land Development Department, Thailand. The Geographical origins as point locations presenting in the map were calculated the centroid of polygons using Geometry tools in program QGIS v.3.40.

The 163 *Bt* strains used in this study were originally isolated from diverse ecological sources across various regions of Thailand (Table 1, Fig. 1) (Attathom et al., 1995; Thaphan, Keawsompong & Chanpaisaeng, 2008; Lertwiriyawonga et al., 2010). Furthermore, *B. thuringiensis* subsp. *aizawai* (XenTari^®^ Biological Insecticide) was used as a reference strain. All the strains were maintained for long-term storage at −20 °C in nutrient broth (Merck, Darmstadt, Germany) supplemented with 30% glycerol in the culture collection of the Department of Entomology, Faculty of Agriculture, Kasetsart University, Thailand.

### Screening of *vip3* gene by polymerase chain reaction

Genomic DNA was extracted from the *Bt* strains using the PureLink™ Genomic DNA Mini Kit (Invitrogen, USA) according to the manufacturer’s instructions. Detection of the *vip3*-type gene was performed using the primer pair Vip3F (5′-ACATCC TCCCTACACTTTCTAATAC-3′) and Vip3R (5′-TCTTCTATGGACCCGTTC TCTAC-3′) (Espinasse et al., 2003), which amplifies a 678 bp product. Polymerase chain reaction (PCR) was carried out in a total reaction volume of 50 µL. Each PCR mixture contained 3 µL of DNA template, 1.25 U of Taq DNA polymerase (invitrogen), 5 µL of 10X reaction buffer, 3 µL of 1.5 mM MgCl_2_, 1 µL of 10 mM each dNTP, and 1 µL of 10 mM of each primer. The PCR cycling conditions were as follows: initial denaturation at 94 °C for 5 min, followed by 30 cycles of denaturation at 94 °C for 1 min, annealing at 45 °C for 45 s, and extension at 72 °C for 2 min. The final extension was performed at 72 °C for 10 min. The amplified products were separated by electrophoresis on a 1% agarose gel. The *Bt* strains confirmed to harbor a *vip3* gene were selected for further determination of insecticidal activity.

### Determination of larvicidal activity of Vip3 protein

#### Insect colonies

Eggs of *S. litura*, *S. exigua*, and *S. frugiperda* obtained from the National Science and Technology Development Agency, Thailand. Newly hatched larvae were gently transferred to a semisynthetic diet composed of dried bean, ascorbic acid, methyl 4-hydroxybenzoate, sorbic acid, B vitamins, wheat germ, yeast extract, and agar (Güney et al., 2019). Feeding activity after hatching was confirmed by direct visual observation of larval movement and feeding marks on the diet surface (Sanané et al., 2021). Larvae that failed to initiate feeding or showed signs of starvation were excluded from subsequent bioassays. The remaining larvae were reared on the diet until they reached the second-instar for use in bioassays (Idrees et al., 2023). Insect colonies were maintained under controlled environmental conditions (25 ± 2 °C, 70 ± 10% relative humidity, and a 16:8 h light:dark photoperiod) in the insectaries at the Research and Lifelong Learning Center on Urban and Environmental Entomology, Thailand. All experimental procedures involving insect specimens adhered to the ethical guidelines established by the Thai Institutional Animal Care and Use Committee (IACUC) and National Research Council of Thailand (NRCT) (license No. ACKU68-AGR-005).

**Table 1:** Geographical origins and samples sources containing *Bacillus thuringiensis* strains in Thailand.

Geographical origins	Source of samples			Total number of strains
	Soil	Stem borer cadaver	Rice bran	
North	15	0	13	28
North-East	17	1	10	28
Central	14	1	15	30
East	16	0	7	23
West	9	0	14	23
South	25	0	6	31
Total (strains)	96	2	65	163

#### Preparation of supernatants from *Bacillus thuringiensis* for screening and quantitation of Vip3

Vip3-positive *B. thuringiensis* strains were inoculated in Luria-Bertani broth (Sigma-Aldrich, St. Louis, MO, USA) and cultured at 37 °C for 16–18 h. Five milliliter of this culture was subsequently inoculated in five mL of Terrific Broth (Fisher Scientific, Waltham, MA, USA), which was then grown for 48 h. The culture was centrifuged at 12,000×g for 10 min at 4 °C, and the resulting supernatant containing Vip3 was collected (Sattar et al., 2008). The supernatant was concentrated by centrifugal ultrafiltration (Vivaspin^®^ 500, Merck), which resulted in the removal of proteins with a molecular weight less than 30 kDa, including cytolytic proteins. Total protein concentration was determined using the Bradford method (Bradford, 1976); a calibration curve was prepared using bovine serum albumin as a standard protein. The standard and test samples were transferred to a 96-well microplate, and the absorbance was measured at 595 nm using a UV-visible spectrophotometer (Bio-Rad).

Toxins, specifically Vip3 and other exotoxins, are secreted into the supernatant during the vegetative phase. Because Vip3 is a heat-sensitive protein, a parallel experiment was conducted to confirm whether the larvicidal activity was due to Vip3 alone and not due to the exotoxins. Autoclaved (121 °C for 15 min) and unautoclaved supernatants were tested using the same procedure based on the rationale that larvicidal activity observed for the autoclaved supernatant would suggest that the toxicity was not caused by Vip3 alone.

#### Larvicidal activity of Vip3 against *Spodoptera* pests

Second-instar larvae of *S. litura*, *S. exigua*, and *S. frugiperda* were used to test the activity of culture supernatants containing secreted Vip3A. This stage was selected for its consistent feeding behavior, ease of handling, and high sensitivity to *Bt* toxins, which collectively enhance experimental sensitivity and reproducibility (Hellmich et al., 2001). Furthermore, second-instar larvae are generally used in toxicity bioassays as they minimize the physiological variability and increased toxin tolerance often observed in later instars (Idrees et al., 2022).

The larvicidal activity was assessed by applying 100 µL of unautoclaved and autoclaved culture supernatant onto the surface of a semisynthetic diet (two cm^2^ multiwell plates). After the supernatant had dried, second-instar larvae of each *Spodoptera* species were added to different wells. Sixty larvae were tested for each *Bt* strain.

All experiments were conducted in triplicate under laboratory conditions (25 ± 2 °C and 70–80% relative humidity). Larvae were considered dead when no signs of movement were observed after they were prodded with a sterile stick (Dulmage et al., 1990). Mortality was further confirmed when larvae failed to respond to repeated stimulation over an extended observation period, allowing for true mortality to be distinguished from thanatosis. Mortality rates were assessed by recording the number of dead larvae 24, 48, and 72 h after the bacterial suspension was applied. The percentage of larval mortality was corrected for the control mortality using the Abbott’s formula (Abbott, 1925).

### Morphology of crystal protein

For examining the morphology of crystal proteins, spore-crystal suspensions were placed on coverslips and air-dried on aluminum mounts. The samples were subsequently coated with a thin layer of platinum using a sputter coater (Quorum Q150R ES). Finally, the dried samples were imaged using a Hitachi SU8020 scanning electron microscope (SU8020 FE-SEM; HITACHI Ltd., Chiyoda City, Tokyo, Japan) at an acceleration voltage of 5 kV.

### Biochemical characterization

The biochemical characteristics of *B. thuringiensis* strain were examined using the API 50CHB system (BioMérieux, Mérieux Étoile, France). A single colony from each strain was selected and emulsified in inoculating fluid for subsequent inoculation in a microplate test. The inoculum was prepared for a specified transmittance using a turbidity meter, as specified in the user guide by Logan & Berkeley (1984).

### Detection of the *cry* gene

PCR was performed using specific primers to identify the following lepidopteran toxin-coding genes: *cry1Aa*, *cry1Ab*, *cry1Ac*, *cry1Ad*, *cry1Ae*, *cry1B*, *cry1C*, *cry1D*, *cry1E*, *cry1F*, *cry1G*, *cry1H*, *cry1I*, *cry1J*, *cry1K*, *cry2A*, *cry5*, *cry12*, and *cry13A* (Juarez-Perez, Ferrandis & Frutos, 1997; Bravo et al., 1998; Masson et al., 1998; Ejiofor & Johnson, 2002; Porcar et al., 2014). The amplification was performed under the following conditions: denaturation at 94 °C for 5 min, followed by 30 cycles of amplification, with each cycle comprising denaturation at 94 °C for 1 min, annealing, at a temperature dependent on the primer set used, for 45 s, and extension at 72 °C for 2 min. PCR products were analyzed by 1% agarose electrophoresis. PCR product for each *cry* gene was purified using the QIAquick^®^ PCR purification kit (Qiagen, Hilden, Germany), and sent to Macrogen (Seoul, South Korea) for DNA sequencing.

### Complete sequence of the *vip3* gene

Genomic DNA from the high-toxicity *Bt* strain was used for amplifying the open reading frame of the *vip3* gene. This was achieved using specific primers designed for this purpose (Şahin et al., 2018): forward (5′-CGCGGATCCATCGAAGGTCGTATGAACAAG AATAATACTAAAT-3′) and reverse (5′-AAGGAAAAAGCGGCCGCTTACTTAATAGAG ACATCGTAA-3′). The PCR mixture comprised 25 µL of 2X PCRBIO Taq Mix Red (PCR Biosystems, UK), 2 µL of each primer (10 pmol/µL), 10 µg of genomic DNA template, and nuclease-free water in a final volume of 50 µL. PCR amplification was performed using a T100™ Thermal Cycler (Bio-Rad) under the following conditions: initial denaturation at 96 °C for 4 min, followed by 35 cycles of denaturation at 96 °C for 40 s, annealing at 50 °C for 1 min, and extension at 72 °C for 2 min, with a final extension at 72 °C for 5 min. The resulting PCR products were analyzed by electrophoresis on a 1.5% agarose gel stained with RedSafe (iNtRON Biotechnology, Seongnam-si, South Korea) in 0.5X TAE buffer (0.02 M Tris base, 0.01 M acetic acid, 0.5 mM EDTA pH 8.0) at 100 V for 30 min. A GeneRuler 100 bp plus DNA ladder (Thermo Scientific) was used as a molecular size marker. The DNA bands were visualized using a Gel Doc system (Syngene).

The amplified PCR products were subjected to Sanger sequencing. The obtained sequences were compared with those in the GenBank database to determine the percentage of similarity (% Max Identity) and sequence variation. ClustalW (http://www.ebi.ac.uk/clustalw/) was used for multiple sequence alignment of the partial *vip3* sequence. The resulting nucleotide sequence served as a template for primer design to facilitate gene walking and obtain the complete gene sequence.

### Expression and purification of Vip3 in *Escherichia coli*

The complete *vip3* sequence was synthesized, cloned, and purified by GenScript (Piscataway, NJ, USA). *Escherichia coli* BL21(DE3) competent cells were transformed with the pET30a(+) vector and cultured overnight at 37 °C on LB agar containing kanamycin (30 µg/mL). The transformed bacterial colonies were selected and inoculated in LB medium containing 30 µg/mL kanamycin and grown until the optical density at 600 nm (OD 600) reached 0.6–0.8. Protein expression was induced with 1.0 mM isopropyl-β-D-thiogalactopyranoside (IPTG) at 15 °C for 16 h. Cell pellets were obtained by centrifugation at 6,000 × g for 20 min at 4 °C and sonicated in lysis buffer (50mM Tris, 300mM NaCl, and 7.5% glycerol [pH 8.0], and 1 mg/mL lysozyme). The lysate was clarified by centrifugation at 12,000 × g for 30 min at 4 °C, and the supernatant was purified using a Ni-NTA column.

The target protein was eluted with an elution buffer (50 mM Tris–HCl, 150 mM NaCl, 50 mM imidazole, pH 8.0) and subsequently dialyzed against storage buffer (50 mM Tris–HCl, 150 mM NaCl, 10% glycerol, pH 8.0). The purity and amount of Vip3 were determined by electrophoresing on a sodium dodecyl sulfate-polyacrylamide gel (Laemmli, 1970), followed by staining with Coomassie Brilliant Blue R-250 (Sigma–Aldrich).

### Evaluating the toxicity of Vip3 insecticidal protein

To investigate the potential of the purified Vip3, the LC_5__0_ and LC_90_ were determined using surface contamination assays at five concentrations: 100, 1,000, 2,500, 5,000, and 10,000 ng/mL. The Vip3Aa94 protein was diluted with 0.01 M phosphate-buffered saline, which also served as the control. For the assays, 100 µL of each concentration was applied onto an artificial diet in 1.9 cm^2^ multiwell plates. Once the protein solution was completely absorbed, a single of second-instar larvae was placed into each well, resulting in 24 larvae for each concentration. The bioassay was conducted with three independent replicates, reaching a total sample size of 72 larvae per treatment. The experiment of each insect pest (*S. litura*, *S. exigua*, and *S. frugiperda*) was replicate three times under the aforementioned laboratory conditions. Larval mortality was recorded daily for three consecutive days (at 24 h interval). The accumulated number of dead larvae for each Vip3 protein concentration was used to calculate the corrected mortality using Abbott’s formula (Abbott, 1925).

### Phylogenetic analysis of *vip3* sequences

Phylogenetic relationship among *Bt* strains was inferred using maximum likelihood (ML) and Bayesian inference (BI) approaches. Before constructing the phylogenetic trees, the best-fit evolution models of nucleotide substitution were selected using the Kakusan4 program (Tanabe, 2007). The selection was based on the Akaike Information Criterion (AIC) (Akaike, 1974) for ML analysis and the Bayesian Information Criterion (BIC) (Schwarz, 1978) for BI analysis. Maximum likelihood (ML) analyses were performed using IQ-TREE v2.2.2.7 (Minh et al., 2020). Nodal support was assessed with 10,000 ultrafast bootstrap replicates (UFBoot) (Hoang et al., 2018). The resulting phylogenetic trees were visualized and edited using FigTree v1.4.4. Branches with bootstrap values of at least 70% were considered to be well-supported clades, following the criteria of Huelsenbeck & Hillis (1993).

Bayesian phylogenetic inference was conducted using MrBayes version 3.2.6 (Ronquist et al., 2012), employing the Metropolis-coupled Markov Chain Monte Carlo algorithm. Two independent runs, each with four chains, were executed in parallel for 100,000 generations, with trees sampled every 1,000 generations. The first 25% of the sampled trees were excluded as “burn-in” to ensure the stabilization of the posterior distribution. A majority-rule consensus tree topology was generated from the remaining trees to assess the phylogenetic topology and posterior probabilities of the clades (Huelsenbeck & Ronquist, 2001).

Nodes with a posterior probability value greater than 0.94 were considered strongly supported (Larget & Simon, 1999). To root the phylogenetic tree, two *vip* sequences from *Bt* strains, strain 376 vip1 (GenBank accession number GU992203.1) and Sbt009 vip4 (accession no. HM044666.1) were included as outgroup taxa. These sequences, which were derived from more distantly related clades, were selected to provide appropriate rooting and context for interpreting the relationships between the *vip3* genes.

### Statistical analysis

The toxicity of *B. thuringiensis* against Spodoptera species was evaluated based on the corrected mortality (%) among 10 *Bt* strains using one-way analysis of variance at a 99% significance level. Tukey’s post-hoc test was used to identify the *Bt* strains that showed statistically significant differences in toxicity. Mortality rates were expressed as the mean ± SE%. All statistical analyses were conducted using the Jamovi software (version 2.3.28). The estimation of LC_50_ values was conducted using Probit analysis (Finney, 1971) in IBM SPSS software (version 25). Differences between LC_50_ values were considered statistically significant when their fiducial limits did not overlap.

## Results

### PCR-based screening of *vip3*

Genomic DNA from 163 *Bt* strains was subjected to PCR amplification using screening primers designed from conserved regions within the *vip3* gene. The expected size of the PCR product was approximately 678 bp. Seventeen (10.43%) *Bt* strains—JC1, JC20, JC39, JC43, JC68, JC81, JC128, JC235, JC247, JC353, JC354, JC356, JC397, JC399, JC400, JC406, and JC414—yielded an amplification product for *vip3*, whereas the remaining 146 (89.57%) strains did not. Among the 17 *vip3*-positive *Bt* strains, eight (47.06%) were isolated from soil samples and rice bran, whereas one strain was obtained from an insect source. Most *vip3*-positive strains were geographically isolated from northern Thailand.

### Larvicidal activities of Vip3 against *Spodoptera* pests

The larvicidal activity of the 17 *Bt* strains against the second-instar larvae of *S. litura*, *S. exigua*, and *S. frugiperda* was evaluated using an artificial diet that included Vip3 containing supernatant. Of the 17 *vip3*-positive *Bt* strains, seven (JC1, JC39, JC68, JC247, JC354, JC400, and JC406) did not cause mortality in *S. litura*, *S. exigua*, and *S. frugiperda.* These results were consistent with those for the standard strain *B. thuringiensis* subsp. *aizawai*. Ten *vip3*-positive *Bt* strains, with protein concentrations, as determined using the Bradford method, ranging from a low of 1.59 mg/mL in strain JC20 to a high of 1.86 mg/mL in JC81, were found to exhibit larvicidal activity. Despite having a lower protein concentration, JC20 exhibited a significantly higher corrected mortality rate than the other *vip3*-positive *Bt* strains (*P* < 0.001). It achieved corrected mortality rates of 98.30 ± 1.67% against *S. litura* and both 100% against *S. exigua* and *S. frugiperda*. JC81 and JC397 were the second and third most effective strains, with high corrected mortality rates ranging from 70.00% to 78.30% against the three *Spodoptera* pests. In contrast, JC43 caused the lowest mortality, with rates of 20.00 ± 2.46% for *S. litura*, 35.00 ± 5.00% for *S. exigua*, and 35.00 ± 6.09% for *S. frugiperda*. Based on its superior performance, JC20 was selected for further investigation (Table 2). The autoclaved supernatants from the 17 *vip3*-positive *Bt* strains did not cause insect mortality. This indicated that the larvicidal activity observed in the non-autoclaved supernatants was due to a heat-sensitive molecule, likely Vip3 Protein.

### Morphology and biochemical characterization of crystal protein

**Figure 2:**
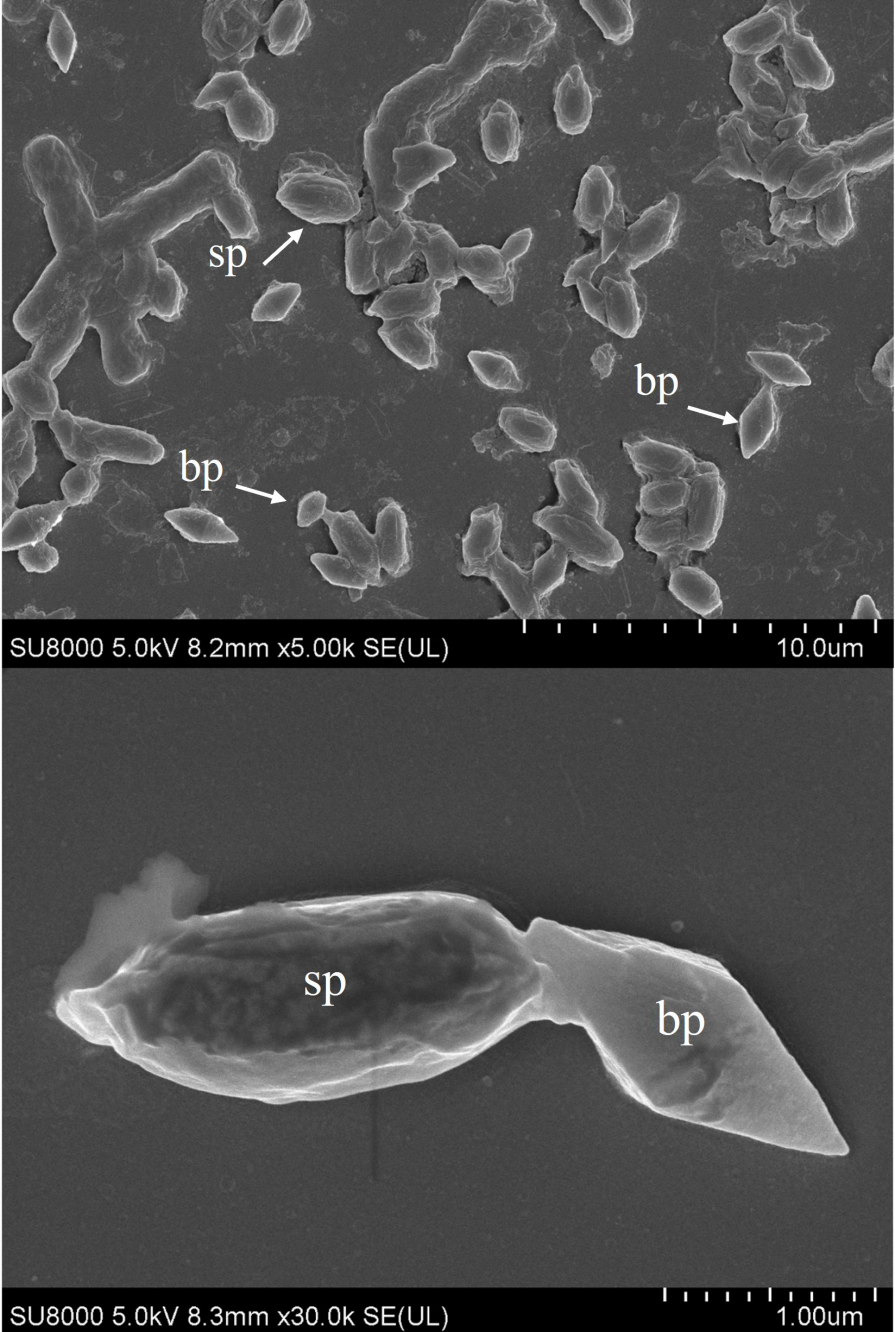
Scanning electron microscopy of spores and crystal proteins from *Bacillus thuringiensis* strains JC20. Abbreviations: bp, bi-pyramidal crystal; sp, spore.

Scanning electron microscopy revealed that JC20 was a rod-shaped cell capable of producing oval spores and bipyramidal crystals with distinct small and large forms (Fig. 2). The biochemical profiles of JC20 are presented in Table 3. JC20 exhibited slight differences from *B. thuringiensis* subsp. *aizawai*. It is capable of fermenting L-arabinose sugar but not D-raffinose and beta-gentiobiose. Based on the API kit database and these biochemical characteristics, JC20 was identified as *B. thuringiensis* with 99.9% shared identity.

### Determination of the *cry* gene content

PCR analysis of JC20 using specific primers for *cry* genes revealed the presence of *cry1D* and *cry2A*. All PCR products were cloned, sequenced, and subjected to Basic Local Alignment Search Tool (BLAST) using the National Center for Biotechnology Information (NCBI) database. Sequence comparisons with other *cry* genes in the database showed that the *cry1D* and *cry2A* sequences from JC20 were highly homologous to the sequences of *cry1Db* (AF358862.1) and *cry2Ab* (JN226103.1), with identities of 99.62% and 95.82%, respectively.

**Table 2:** *Bacillus thuringiensis* caused a corrected mortality rate in *Spodoptera* species after 72 h of exposure to an artificial diet containing a Vip3 protein supernatant.

*Bt* strain	Protein concentration (mg/mL)	Corrected Mortality (%) (mean ± SE)^*^		
		*S. litura*	*S. exigua*	*S. frugiperda*
JC20	1.59	98.30 ± 1.67a	100.00 ± 0.00a	100.00 ± 0.00a
JC43	1.61	20.00 ± 2.46d	35.00 ± 5.00c	35.00 ± 6.09cd
JC81	1.86	70.00 ± 3.81b	78.30 ± 2.97b	73.30 ± 2.84b
JC128	1.62	53.30 ± 5.69bc	56.70 ± 4.82bc	36.70 ± 4.82cd
JC235	1.77	23.30 ± 4.82d	41.70 ± 3.86c	18.30 ± 4.58d
JC353	1.80	23.30 ± 5.95d	40.00 ± 4.26c	40.00 ± 4.92cd
JC356	1.71	23.30 ± 5.41d	40.00 ± 5.50c	41.70 ± 5.75c
JC397	1.67	65.00 ± 5.57b	63.30 ± 3.33b	56.70 ± 4.14bc
JC399	1.83	43.30 ± 5.95bcd	40.00 ± 6.03c	43.30 ± 5.41c
JC414	1.65	58.30 ± 7.16bc	65.00 ± 4.35b	41.10 ± 5.75c
ANOVA	F	25.8	23.7	23.4
	df	9	9	9
	*p*	<.001	<.001	<.001

**Notes.**

* Different lowercase letters after the means of corrected mortality (%) within each *Spodoptera* species indicate a significant difference (*p* < 0.01) by Tukey’s *post hoc* test.

**Table 3:** Biochemical profiles of *Bacillus thuringiensis* strains JC20 compared with the reference strain *Bacillus thuringiensis* subsp. *aizawai*.

Test	JC20	*aizawai*	Test	JC20	*aizawai*
Glycerol	+	+	Salicin	+	+
Erythritol	–	–	Cellobiose	+	+
D-Arabinose	–	–	Maltose	+	+
L-Arabinose	+	–	Lactose	–	–
Ribose	+	+	Melibiose	–	–
D-xylose	–	–	Sucrose	–	–
L-xylose	–	–	Trehalose	+	+
Adonitol	–	–	Inulin	–	–
Beta-Methylxyloside	–	–	Melezitose	–	–
Galactose	–	–	D-Raffinose	–	+
D-Glucose	+	+	Starch	+	+
D-Fructose	+	+	Glycogen	+	+
D-Mannose	–	–	Xylitol	–	–
L-Sorbose	–	–	Beta-Gentiobiose	–	+
Rhamnose	–	–	D-Turanose	–	–
Dulcitol	–	–	D-Lyxose	–	–
Inositol	–	–	D-Tagatose	–	–
Mannitol	–	–	D-Fucose	–	–
Sorbitol	–	–	L-Fucose	–	–
Alpha-Methyl-D-mannoside	–	–	D-Arabitol	–	–
Alpha-Methyl –D-glucoside	–	–	L-Arabitol	–	–
*N*-Acethylglucosamine	+	+	Gluconate	+	+
Amygdalin	+	+	2-Ketogluconate	–	–
Arbutin	+	+	5-Ketogluconate	–	–
Esculin	+	+			

**Notes.**

Key + positive reaction – negative reaction

### Complete sequence of *vip3*

JC20 was selected for complete sequencing of its *vip3* gene because of its high toxicity against *Spodoptera* species in bioassays with the supernatant. The sequence of the primer designed for PCR walking was 5′-AAC AAG TGG CAG TGA AGT AGG-3′. The PCR product for each *vip3* gene was cloned and sequenced to obtain a complete sequence of 2,370 bp. Sequence comparison with other *vip3* genes using the BlAST-N program in the NCBI database showed that these sequences have a high homology to the Vip3Aa17 sequence, with approximately 99.9% identity. The nucleotide sequence was deposited in GenBank (accession number: PV339976). The protein was named Vip3Aa94 by Neil Crickmore (The Bacterial Pesticidal Protein Resource Center database, https://bpprc.org/).

### Expression and purification of Vip3 in *E. coli*

**Figure 3:**
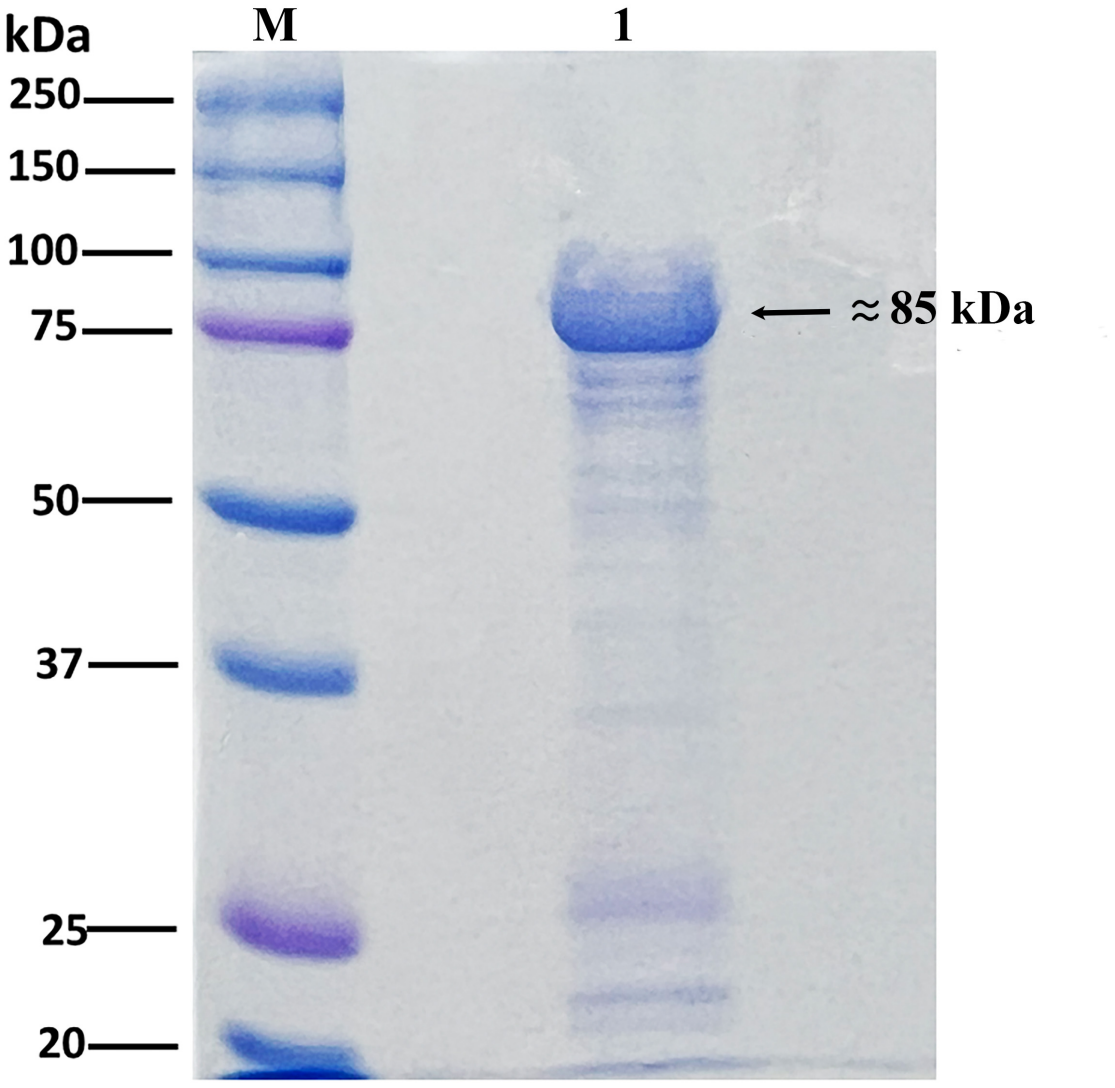
SDS-PAGE gel showing protein expression of Vip3Aa94. Lane M: Precision Plus Protein Standards (Bio-Rad). Lane 1: Vip3 protein expressed and purified using a Ni-NTA column. The arrow indicates the putative vegetative insecticidal protein (Vip3) at ∼85 kDa.

The protein encoded by *vip3Aa94* was expressed in *E. coli* BL21 (DE3) following IPTG induction. The molecular weight of the expressed protein was approximately 90 kDa, as expected (Fig. 3), which corresponded to that of Vip3.

### Evaluating the toxicity of Vip3Aa94 insecticidal protein

**Figure 4:**
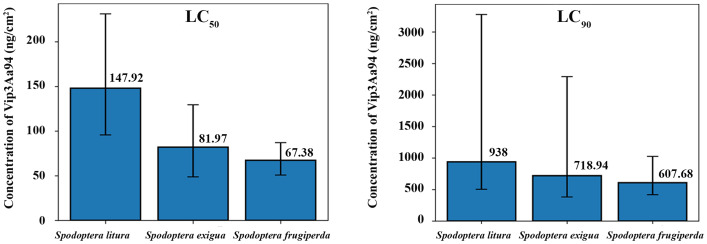
Lethal concentrations of Vip3Aa94 insecticidal protein from *Bacillus thuringiensis* JC20, against three Spodoptera species at 3 days post-application.

The toxicity of Vip3Aa94 insecticidal protein from *B. thuringiensis* JC20 against three species of *Spodoptera* was examined using probit analysis. The protein Vip3Aa94 exhibited the highest toxicity to *S. frugiperda*, with LC_50_ and LC_90_ value of 67.38 ng/cm^2^ and 607.68 ng/cm^2^, respectively. In contrast, *S. litura* was the least susceptible, with LC_50_ and LC_90_ value of 147.92 ng/cm^2^ and 938 ng/cm^2^, respectively. For the LC_50_, the 95% fiducial limits of *S. frugiperda* were non-overlapping with those of *S. lituta*, showing that the toxicity against *S. frugiperda* was higher than against *S. litura*. Additionally, no heterogeneity effect of Vip3Aa94 was observed among individual *S. frugiperda* larvae (*χ*2 = 21.05, *p* = 0.072), which indicates that the tested population responded uniformly to the toxin (Fig. 4, Table S1).

### Phylogenetic analysis of *vip3* sequences

**Figure 5:**
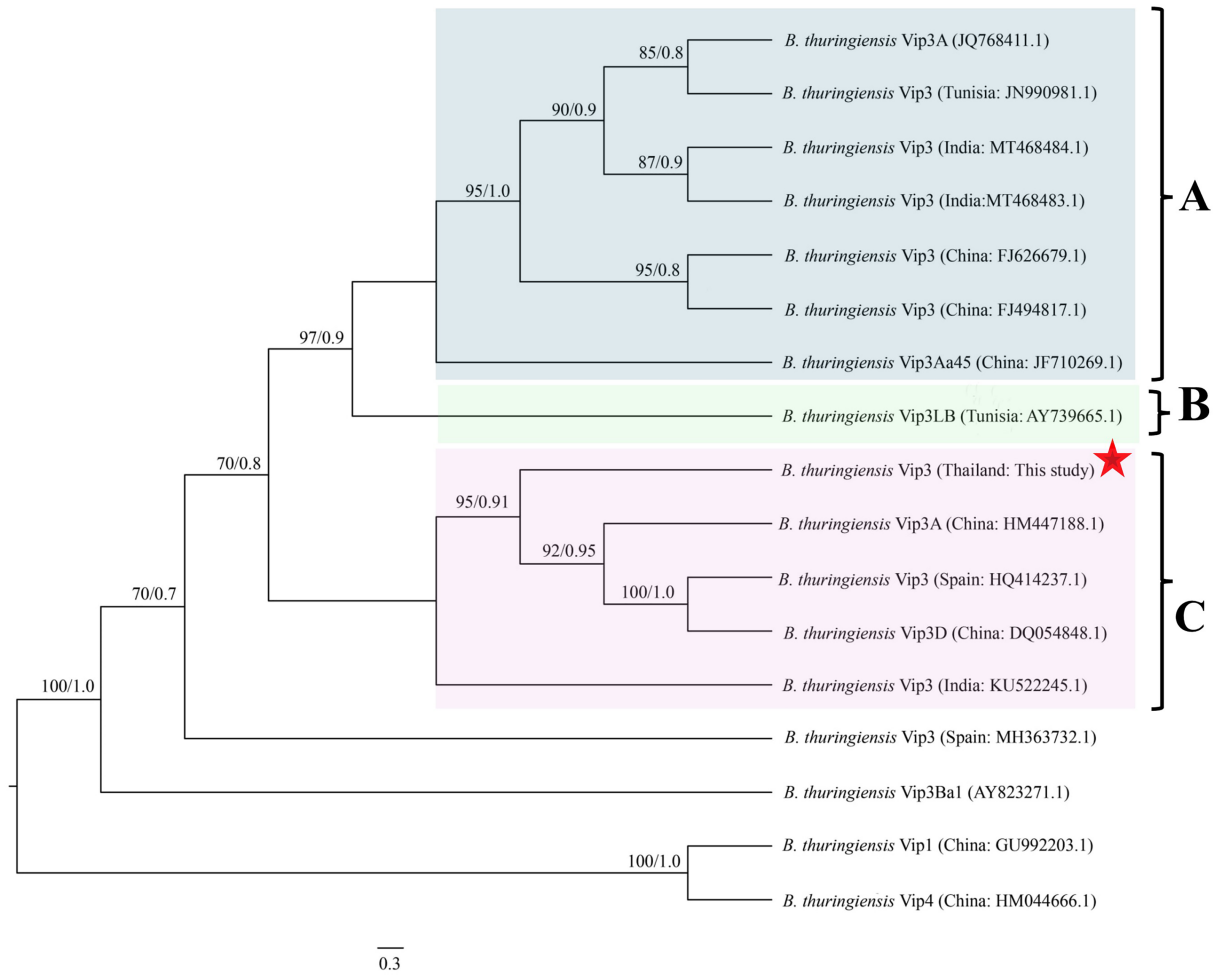
Phylogenetic relationships among Vip3 protein gene sequences of *Bacillus thuringiensis* inferred using the maximum likelihood (ML) method. Support values at nodes are shown as Bayesian posterior probabilities (BI) and ML bootstrap values (BS), presented as BI/ML. The scale bar represents the number of substitutions per site.

Phylogenetic analysis of the *vip3Aa94* nucleotide sequences revealed the formation of three well-defined clades, designated Clusters A, B, and C (Fig. 5). These clusters were moderately to strongly supported by Bayesian posterior probability values ranging from 0.7 to 0.9. The *vip3Aa94* sequence from *B. thuringiensis* strain JC20 (GenBank number PV339976) was assigned to Cluster C, which was further divided into two subclades, indicating a more complex evolutionary structure within this lineage.

Interestingly, JC20 showed phylogenetic divergence from the sequences grouped in Clusters A and B. Within Cluster C, JC20 *vip3* was positioned distinctly apart from the Indian isolates (*e.g.*, KU522245.1). It was rather more closely related to sequences from China (HM447188.1) and Spain (HQ414237.1), as well as to *vip3D* sequences from China (DQ054848.1). This pattern is indicative of a possible regional association or horizontal gene transfer event that could contribute to the observed genetic similarity among geographically distant strains.

## Discussion

Thailand is recognized for harboring a high diversity of *Bt* strains (Thaphan, Keawsompong & Chanpaisaeng, 2008; Lertwiriyawonga et al., 2010). Globally, numerous *B. thuringiensis* serovars have been described, including *thailandensis* and *chanpaisis*, which were originally isolated in Thailand (Lecadet et al., 1999; Rajchanuwong, Chanpaisaeng & Kaewsompong, 2019). Previous studies demonstrated substantial diversity in the cry gene content of Thai *Bt* strains, harboring toxins effective against lepidopteran and dipteran pests. The discovery of the less widespread *cry32* gene, specifically toxic to mosquito larvae, further highlights this regional genetic richness (Thammasittirong & Attathom, 2008; Boonmee, Thammasittirong & Thammasittirong, 2019; Rajchanuwong, Chanpaisaeng & Kaewsompong, 2019; Rajchanuwong et al., 2025).

Vip3 proteins differ from Cry proteins in their binding sites and modes of action and are effective against a wide range of lepidopteran pests (Ferré et al., 2023). Although *vip* 3 genes have been extensively investigated in *Bt* collections worldwide, reports from Thailand remain limited, underscoring the importance of further characterization of v*ip* 3-harboring Thai *Bt* strains.

This study screened 163 *Bt* strains collected from various regions and ecological sources in Thailand. Of these, only 17 strains (10.43%) tested positive for the *vip* 3 gene. This relatively low frequency indicated that *vip* 3 genes are present but not widespread in Thai *Bt* populations. This pattern is consistent with previous reports from Thailand (Thaphan, Keawsompong & Chanpaisaeng, 2008; Boonmee, Thammasittirong & Thammasittirong, 2019; Hemthanon, Promdonkoy & Boonserm, 2023).

Considerable variation in *vip* 3 gene frequency has been reported among *Bt* collections worldwide, ranging from high prevalence to low or undetectable levels (Table 4). Such variation is influenced by multiple biological factors and environmental factors, including horizontal gene transfer, ecological conditions, and geographic isolation. *Bacillus thuringiensis* strains are known to exchange genetic material, such as *cry* and *vip* genes, *via* horizontal gene transfer (González Jr, Brown & Carlton, 1982; Ehling-Schulz, Lereclus & Koehler, 2019; Hinnekens et al., 2022). However, limitations in gene exchange—imposed by geography, environmental conditions, and transfer mechanisms—can lead to the accumulation of distinct *cry/vip* gene combinations within regional *Bt* populations over time (Thomas et al., 2000; Vilas-Boas et al., 2002; Patel, Purani & Ingle, 2013; Hinnekens et al., 2022). Consequently, environmental factors and geographic isolation may further shape the distribution and diversity of *Bt* toxin genes by favoring strains that produce Cry/Vip toxins effective against locally prevalent pests (Uribe, Martinez & Ceron, 2003; Patel, Purani & Ingle, 2013).

**Table 4:** *vip3*—type gene distribution in *Bacillus thuringiensis* isolates from different countries.

Country of Bt collection	Gene size (bp)	Vip3 Gene frequency (%)	Number of isolates/strains	Ecological source	References
Thailand	678	10.42	163	Soil and rice bran	This study
Thailand	1,591	47.55	511	Soil	Boonmee, Thammasittirong & Thammasittirong (2019)
Thailand		21.42	42	Not mentioned	Hemthanon, Promdonkoy & Boonserm (2023)
Sri Lanka	1,029	42	21	Soil	Baragamaarachchi et al. (2019)
Turkish	1,395	23	80	Soil, fruits, and fig leaves	Şahin et al. (2018)
India	1,400	40	15	Soil from tea and rice field	Rabha, Acharjee & Sarmah (2018)
Argentia	608	91.3	268	Soil, spider web, leaves, dust, dead larvae	Sauka & Benintende (2017)
India	700	43.18	44	Lake sediments, forest soil, and maize field	Lone et al. (2016)
India	675	5.33	150	Soil/infected insects	Rangeshwaran et al. (2016)
Tunisia	670–2,370	30	212	Soil	Sellami et al. (2013)
Spain	364, 444	14.5	400	Soil samples, barn dust, and aquatic environments.	Palma et al. (2013)
China	364, 444	67.4	2,134	Soil from Mountain, Forest Farmland and snowcapped mountain	Yu et al. (2011)
Spain and Bolivia	1,621	48.9	507	Soil, dust, grain	Hernández-Rodríguez et al. (2009)
Australia	1,621	87	187	Soil, bird nest, and grain dust	Beard et al. (2008)
Iran	1,000	82.6	70	Soil, leaf samples, and dead insects	Seifinejad et al. (2008)
Tunisia	419	30	259	Soil	Mesrati, Tounsi & Jaoua (2005)
France and 31 countries of 5 continents	678	52.8	125	Soil, plants, animal waste, dust, insects, *etc.*	Espinasse et al. (2003)
Brazil	150, 1,210	100	12	Soil	Loguercio et al. (2002)
India	700	2	49	Soil	Selvapandiyan et al. (2001)

In addition to biological factors and environmental factors, methodological variation is recognized as a major contributor to differences in reported *vip* 3 gene frequencies. Differences in primer design, PCR conditions, and sampling strategies can substantially influence detection outcomes, thereby limiting direct comparisons among surveys (Porcar & Juárez-Pérez, 2003). Even when similar primers and PCR conditions are applied, variation in *vip* gene frequencies has been observed within the same geographic region, highlighting the combined influence of technical factors and underlying biological diversity (Boonmee, Thammasittirong & Thammasittirong, 2019).

In this study, *vip* 3-positive *Bt* strains were more frequently associated with isolates originating from soil- and rice bran–derived samples in northern Thailand. This distribution is consistent with previous reports identifying northern and northeastern Thailand as regions with relatively higher occurrence of *vip* 3 (Boonmee, Thammasittirong & Thammasittirong, 2019). Collectively, these findings indicated that *vip* 3 gene distribution reflects an interaction between ecological context and research methodology, underscoring the importance of region-specific screening to discovery potent *Bt* strains for integrated pest management.

The larvicidal activity of the 17 *vip3*-positive *Bt* strains was assessed against *S. exigua*, *S. litura*, and *S. frugiperda*, revealing marked variation in toxicity among the isolates. Notably, JC20 proved to be highly effective, causing 100% corrected mortality against *S. exigua* and *S. frugiperda* and 98.30% against *S. litura*. The high potency observed in this study exceeds the 90% mortality in *S. exigua* reported for the *Bt* 6A supernatant containing Vip3Aa (Şahin et al., 2018), as well as the 86.6% and 83.3% mortality rates against *S. littoralis* achieved by Vip3 proteins from *B. thuringiensis* BnBt and MnD isolates, respectively (Güney et al., 2019). Consequently, JC20 exhibited substantially higher insecticidal activity than the other *vip* 3-positive strains, identifying it as a highly promising *Vip3*-producing *Bt* strain.

The *vip* 3 gene from JC20 was deposited in GenBank (accession no. PV339976) and designated as Vip3Aa94 by the Bacterial Pesticidal Protein Resource Center (Crickmore et al., 2025). The Vip3Aa94 protein exhibited strong insecticidal activity against second-instar larvae of *S. frugiperda, S. exigua, and S. litura*, as evidenced by low LC_5__0_ values. When compared using standardized LC_5__0_ units, Vip3Aa94 from JC20 demonstrated higher or comparable toxicity than several known variants. Notably, Vip3Aa94 was more effective than Vip3Ab, Vip3Aa16, Vip3Aa45, Vip3Aa58, and Vip3Aa59, all of which generally exhibited higher LC_5__0_ values against neonate *S. exigua* than those observed for Vip3Aa94 against the older second-instar larvae in this study (Chakroun et al., 2012; De Escudero et al., 2014; Palma et al., 2013; Baranek et al., 2015). Similarly, Vip3Ab exhibited lower activity against neonate *S. frugiperda* (De Escudero et al., 2014) than Vip3Aa94. Regarding later larval stages, other Vip3 proteins have also shown reduced insecticidal activity against *S. exigua* and *S. litura* (Song et al., 2016; Nutaratat et al., 2023). Such disparities in larval susceptibility are well documented; later-instar lepidopteran larvae generally exhibit increased tolerance to *Bt* toxins compared with early instars (Gujar et al., 2007; Valadez-Lira et al., 2012; George & Crickmore, 2012; Janmaat, Bergmann & Ericsson, 2014). Accordingly, the lower LC_5__0_ values observed in this study demonstrated the potent insecticidal activity of Vip3Aa94, establishing JC20 as a primary candidate for further molecular characterization and development in lepidopteran pest management.

Seven *Bt* strains did not cause mortality in *S. litura*, *S. exigua*, or *S. frugiperda*, although *vip3* was detected in them. This was possibly due to the use of general primers for *vip3* detection. Currently, 140 Vip3 proteins have been described (Crickmore et al., 2025) and classified into three subfamilies: Vip3A (Estruch et al., 1996), Vip3B (Rang et al., 2005), and Vip3C (Palma et al., 2012). Vip3A proteins are known for their insecticidal activity against a wide variety of lepidopterans, including species that are less susceptible to some Cry1A proteins (*e.g.*, *Agrotis ipsilon*, *S. exigua*, and *S. frugiperda*) (MacIntosh et al., 1990; Estruch et al., 1996). In contrast, Vip3Ba1 caused significant growth delays but no larvicidal effect against *Ostrinia nubilalis* and *Plutella xylostella* (Rang et al., 2005). Preliminary bioassays of Vip3C also showed low activity, causing less than 30% mortality in *S. exigua* and *S. frugiperda* after 10 days at 4 µg/cm^2^ (Palma et al., 2012). Therefore, the observed lack of mortality in the seven *B. thuringiensis* strains indicated that the detected *vip3* genes did not belong to the *vip3A* gene family.

Besides the potent insecticidal activity of its Vip3Aa94 protein, molecular analysis revealed that strain JC20 harbors the *cry1D* and *cry2A* genes, further increasing its value for lepidopteran pest control. Due to their broad-spectrum activity against various insect pests, particularly lepidopteran species, Cry1D and Cry2A are considered valuable components in insect resistance management strategies (Bravo et al., 2004; Zhang et al., 2007; Hernández-Martínez, Ferré & Escriche, 2008; Sasaki et al., 1997; Alcantara et al., 2004). Cry1D is especially notable for its efficacy against *Spodoptera* species, including insects that have developed resistance to Cry1A toxins, and is among the few *δ*-endotoxins active against the highly damaging and Cry-tolerant *S. littoralis* (Hernández-Martínez, Ferré & Escriche, 2008; Bergamasco et al., 2013). Cry2A proteins are also recognized for their wide spectrum of toxicity against insects in the orders Lepidoptera and Diptera and for possessing modes of action distinct from other Cry proteins (Walters & English, 1995; Bravo et al., 2004; Zhang et al., 2007; Van Frankenhuyzen, 2009). Accordingly, JC20 harboring Cry and Vip3 proteins can enhance toxicity against lepidopteran larvae due to their distinct structures, stabilities, and modes of action. While Vip proteins are secreted during the vegetative growth phase and are relatively unstable, *cry* genes accumulate as crystalline inclusion bodies that exhibit superior structural and environmental stability (Federici, Park & Sakano, 2006; Syed et al., 2020; Tetreau et al., 2021).

Previous studies have shown that Vip3 proteins are synthesized and secreted by *Bt* as full-length proteins with molecular weights of approximately 88–90 kDa (Estruch et al., 1996; Lee et al., 2003; Palma et al., 2014). Consistent with this study, the Vip3Aa94 protein from JC20 was successfully expressed in *E. coli* and exhibited an apparent molecular weight close to that expected for Vip3 proteins, confirming its correct expression and structural integrity.

Phylogenetic analyses have previously revealed substantial diversity within the *vip3* gene family, reflecting the evolutionary complexity of insecticidal protein genes in *Bt*. In this study, *vip3* gene sequences clustered into three well-supported phylogenetic groups, consistent with earlier reports describing diversification driven by biogeographic distribution and ecological specialization (De Maagd, Bravo & Crickmore, 2001; Raymond et al., 2010; Gupta, Kumar & Kaur, 2021). Notably, JC20 carrying the *vip3Aa94* gene was positioned within Cluster C, which comprised genetically distinct sublineages including isolates from geographically distant regions. This pattern suggests that the *vip3Aa94* gene may have arisen through evolutionary processes such as geographic isolation, local adaptation to specific insect hosts, or historical horizontal gene transfer events.

The distinct phylogenetic placement of JC20 within Cluster C highlights its potential as a novel source of Vip3 toxins with unique genetic features. Such genetic divergence may contribute to differences in insecticidal specificity or potency, however further functional studies will be required to determine whether sequence variation in Vip3Aa94 protein translates into novel bioactivity or enhances efficacy in pest management.

Although Vip3A proteins exhibit strong activity against lepidopteran pests, their commercial application is often limited by high production costs and protein instability, restricting their current use primarily to transgenic plants (Syed et al., 2020). In Thailand, where the cultivation of transgenic crops is strictly regulated, *Bt* strains like JC20 provide an effective alternative for pest control by combining powerful Vip3A proteins with stable Cry toxins. However, the practical application of these proteins remains challenging due to the limited environmental persistence of Vip3A compared to Cry toxins. In this context, future work focusing on encapsulation technologies to enhance the environmental stability and field efficacy of Vip3 proteins represents a promising alternative. In addition, exploring potential synergistic interactions between Vip3Aa94 and Cry1D or Cry2A toxins present in JC20 may further improve insect control efficacy and contribute to the development of effective *Bt-* based pest management strategies.

## Conclusions

Among the 163 Thai strains of *B. thuringiensis*, 17 were found to contained the *vip3* gene. Off these, JC20 produced Vip3 protein that was most effective against *S. litura*, *S. exigua*, and *S. frugiperda*. Characterization of JC20 revealed that it produces Vip3Aa94, encoded by a newly identified *vip3* gene, in addition to crystal protein genes *cry1D* and *cry2A*. The combined insecticidal activity of these Cry crystals and Vip3 proteins makes JC20 a promising candidate for developing new biopesticides.

##  Supplemental Information

10.7717/peerj.21207/supp-1 Supplemental Information 1 List of *Bacillus thuringiensis* Strains by Geographical Origin and Source

10.7717/peerj.21207/supp-2 Supplemental Information 2 *Bacillus thuringiensis* caused mortality rate in *Spodoptera* species after 72 hours of exposure to an artificial diet containing a Vip3 protein supernatant

10.7717/peerj.21207/supp-3 Supplemental Information 3 Mortality of insects treated with various concentrations of Vip3Aa94 protein

10.7717/peerj.21207/supp-4 Supplemental Information 4 Lethal concentrations of Vip3Aa94 insecticidal protein from *Bacillus thuringiensis* JC20, against three *Spodoptera* species at 3 days post-application

10.7717/peerj.21207/supp-5 Supplemental Information 5 Anova analysis of corrected mortality rate in *Spodoptera exigua* after exposure to a Vip3 protein OMV files can be opened using jamovi (https://www.jamovi.org).

10.7717/peerj.21207/supp-6 Supplemental Information 6 Anova analysis of corrected mortality rate in *Spodoptera litura* after exposure to a Vip3 protein OMV files can be opened using jamovi ( https://www.jamovi.org).

10.7717/peerj.21207/supp-7 Supplemental Information 7 Anova analysis of corrected mortality rate in *Spodoptera frugiperda* after exposure to a Vip3 protein OMV files can be opened using jamovi ( https://www.jamovi.org).

## List of Abbreviations

ANOVA: Analysis of variance
BI: Bayesian inference
Bt: *Bacillus thuringiensis*
CI: Confidence interval
Cry: Crystal protein
DNA: Deoxyribonucleic acid
IPTG: Isopropyl β-D-1-thiogalactopyranoside
IACUC: Institutional Animal Care and Use Committee
kDa: Kilodalton
LC_5__0_: Lethal concentration causing 50% mortality
LC_9__0_: Lethal concentration causing 90% mortality
ML: Maximum likelihood
NCBI: National Center for Biotechnology Information
NRCT: National Research Council of Thailand
OD_6__0__0_: Optical density at 600 nm
PCR: Polymerase chain reaction
SDS–PAGE: Sodium dodecyl sulfate–polyacrylamide gel electrophoresis
SE: Standard error
SEM: Scanning electron microscopy
Vip: Vegetative insecticidal protein

## Additional Information and Declarations

### Data Availability

The following information was supplied regarding data availability: The raw data is available in the Supplemental Files. The sequence is available at NCBI: PV339976.
