# Supplementary material for: A potent Vip3Aa94 protein from Thai Bacillus thuringiensis: molecular characterization and insecticidal potential
Source: PeerJ. 2026 May 27;14:e21207. doi: 10.7717/peerj.21207 (PMC13221986; doi:10.7717/peerj.21207)
Supplement: Supplemental Information 4 [file peerj-14-21207-s004.docx]

**Supplementary Table 1** Lethal concentrations of Vip3Aa94 insecticidal protein from *Bacillus thuringiensis* JC20, against three *Spodoptera* species at 3 days post-application

| Insect test | LC_50_ (ng/cm^2^)  95% Fiducial limits (min-max) | LC_90_ (ng/cm^2^)  95% Fiducial limits (min-max) | Slope±SE | χ2 | p-value |
| --- | --- | --- | --- | --- | --- |
| *Spodoptera lituta* | 147.92  (95.75-231.45) | 938  (501.60-3277.90) | 1.59±0.16 | 36.73 | 0.000 |
| *Spodoptera exigua* | 81.97  (48.78-129.37) | 718.94  (380.64-2292.10) | 1.35±0.13 | 33.98 | 0.001 |
| *Spodoptera frugiperda* | 67.38  (50.69-87.16) | 607.68  (415.25-1024.4) | 1.34±0.13 | 21.05 | 0.072 |
